# Thymidine synthase, thymidine phosphorylase, and excision repair cross-complementation group 1 expression as predictive markers of capecitabine plus cisplatin chemotherapy as first-line treatment for patients with advanced oesophageal squamous cell carcinoma

**DOI:** 10.1038/sj.bjc.6605831

**Published:** 2010-08-10

**Authors:** S Lee, Y H Park, K H Kim, E Y Cho, Y C Ahn, K Kim, Y-M Shim, J S Ahn, K Park, Y-H Im

**Affiliations:** 1 Division of Hematology-Oncology, Department of Medicine, Samsung Medical Center, Sungkyunkwan University School of Medicine, 50 Irwon-dong Gangnam-gu, Seoul 135–710, Korea; 2 Department of Pathology, Samsung Medical Center, Sungkyunkwan University School of Medicine, Seoul, Korea; 3 Department of Radiation Oncology, Samsung Medical Center, Sungkyunkwan University School of Medicine, Seoul, Korea; 4 Department of Thoracic Surgery, Samsung Medical Center, Sungkyunkwan University School of Medicine, Seoul, Korea

**Keywords:** oesophageal cancer, capecitabine, cisplatin, thymidine synthase (TS), thymidine phosphorylase (TP), excesion repair cross-complementation group 1 (ERCC1)

## Abstract

**Background:**

Our purpose was to evaluate thymidine synthase (TS), thymidine phosphorylase (TP), and excision repair cross-complementation group 1 (ERCC1) expression as biomarkers for capecitabine and cisplatin (XP) combination chemotherapy in patients with metastatic oesophageal squamous cell cancer.

**Method:**

A total of 113 patients with metastatic oesophageal squamous cell cancer were treated with XP chemotherapy at the Samsung Medical Center between 2003 and 2007, of whom 72 had available clinical data and paraffin blocks for immunohistochemistry of TS, TP, and ERCC1.

**Results:**

The median age of the 72 patients was 62 years. The overall response rate (RR) was 51.4%. The median progression-free survival (PFS) and overall survival (OS) were 4.2 and 12.0 months, respectively. High expression of TS and TP was associated with a higher RR than was low expression of TS and TP (54.1 *vs* 40.5%, *P*=0.022). Strong ERCC1 expression and a low TS score were identified as unfavourable independent risk factors for PFS (HR 10.71, 95% confidence interval (CI) 2.1–54.7, *P*=0.004 for strong ERCC1 expression; and HR 2.9, 95% CI 1.0–7.9, *P*=0.044 for low TS score). Strong ERCC1 expression was identified as an unfavourable independent risk factor for OS (HR 3.73, 95% CI 1.39–10.0, *P*=0.009).

**Conclusion:**

These data indicate that expression of TS, TP, and ERCC1 may be predictive markers for response and survival in patients with metastatic oesophageal squamous cell cancer receiving XP chemotherapy.

Oesophageal cancer is the eight most frequent malignancy and the fourth highest cause of cancer-related mortality, with almost 500 000 new patients diagnosed annually worldwide (Kamangar *et al*, 2006; Tebbutt *et al*, 2010). The most frequent histological type of oesophageal cancer is squamous cell carcinoma (SCCA), although the proportion of adenocarcinomas in western Europe and the United States is increasing to almost 50%, with no difference in long-term outcomes between the two histological types. Oesophageal cancer is a highly virulent disease with a 5-year survival rate of 10–15% (Lenz *et al*, 1996; Bronckaers *et al*, 2009). At presentation, approximately 50% of patients show distant metastases and the remaining patients who initially present with locoregional disease will eventually develop distant metastases. There is no standard chemotherapeutic regimen for metastatic oesophageal SCCA; hence, various kinds of chemotherapeutic regimens have been investigated in an attempt to prolong survival and improve the quality of life. 5-Fluorouracil (5-FU), cisplatin, and epirubicin are widely used chemotherapeutic agents for gastric and oesophageal cancers worldwide (Kim *et al*, 1993; Ross *et al*, 2002). One of the most commonly used regimens as first-line chemotherapy in metastatic oesophageal cancer is the combination of cisplatin and a 5-FU continuous infusion, with response rates (RRs) ranging from 30 to 40% (Ilzuka *et al*, 1992; Bleiberg *et al*, 1997; Enzinger *et al*, 1999). However, the continuous infusion of 5-FU and cisplatin combination therapy requires indwelling venous access, which may lead to venous thrombosis and sepsis, hence making therapy burdensome to the patient with significant toxicity.

Capecitabine (Xeloda, Hoffmann-La Roche Inc., Basel, CH, USA) is an oral fluoropyrimidine prodrug that is transformed to FU in several steps, the last of which is conversion of 5′-deoxy-5-fluorouridine to FU by thymidine phosphorylase (TP). In patients with advanced oesophagogastric cancer, capecitabine combinations have generally shown good antitumour activity and highlight the potential of capecitabine as a replacement for infusional 5-FU (Cunningham *et al*, 2008; Kang *et al*, 2009). These results have shown that capecitabine is not inferior to FU. In addition, fluropyrimidine-related adverse events were similar in the capecitabine and FU groups. Thus, considering troublesome venous access, capecitabine may be a good substitute for 5-FU.

A phase II trial of our previous study tested the safety and efficacy of capecitabine (1250 mg m^–2^ twice daily for 2 weeks) with cisplatin (60 mg m^–2^ on day 1 in 3-week cycles; capecitabine and cisplatin (XP)) for chemonaive oesophageal SCCA and reported promising results with RRs of 57.8% and a tolerable toxicity profile (Lee *et al*, 2008), suggesting that a combination of capecitabine and platinum is one of the most effective regimens for the treatment of advanced oesophageal cancer. Capecitabine was designed to take advantage of the increased levels of TP observed in tumours as opposed to normal tissue, potentially allowing for selective toxicity in tumours (Van Cutsem *et al*, 2001). Elevated levels of TP are associated with tumour aggressiveness and poor prognosis (Bronckaers *et al*, 2009). 5-FU is then either degraded by dihydropyrimidine dehydrogenase (DPD) or anabolised to fluorodeoxyuridylate, which inhibits thymidylate synthase (TS). Thymidylate synthase, the target enzyme of 5-FU, has been shown to be an independent prognostic marker of 5-FU chemotherapy in gastrointestinal tumours (Lenz *et al*, 1996, 1998; Shirota *et al*, 2001). Meropol *et al* (2006) evaluated TP, TS, and DPD for their ability to predict a response to capecitabine when used in a first-line metastatic setting, in an attempt to identify patients who may have an altered response to a capecitabine/irinotecan regimen. The role of TP and TS to identify predictive markers for the treatment response to XP in oesophageal SCCA has not been defined.

Platinum compounds are additional key drugs in the treatment of oesophageal SCCA. Resistance to platinum agents has been attributed to enhanced tolerance to platinum DNA adducts, decreased drug accumulation, and enhanced DNA repair. Impairment of the DNA repair mechanism is important in the resistance to cisplatin. The destruction of cells by cisplatin requires binding of the drug to DNA and the creation of platinum-DNA adducts. Some of these adducts establish covalent cross-linking between DNA strands, thereby inhibiting DNA replication. Nucleotide excision repair (NER) has a central role in DNA repair and proteins of the NER pathway are thought to repair DNA damage caused by platinum agents. The excision repair cross-complementation group 1 (ERCC1) enzyme has a rate-limiting role in the NER pathway that recognises and removes cisplatin-induced DNA adducts (Olaussen *et al*, 2006).

The significance of TS, TP, and ERCC1 expression in oesophageal SCCA has not been studied in patients who have been treated with capecitabine and platinum combination chemotherapy. In this study, we determined the protein expression of TS, TP, and ERCC1 by immunohistochemical (IHC) staining in pretreatment biopsies of oesophageal tumour tissue specimens from patients who received palliative first-line chemotherapy with XP combination chemotherapy. This study was conducted to evaluate the clinical implications of these proteins as biomarkers, which could predict the outcome of XP chemotherapy.

## Patients and Methods

### Patients

A total of 113 patients with recurrent or metastatic oesophageal SCCA were treated with XP chemotherapy at the Samsung Medical Center between July 2003 and December 2007, of whom 72 had clinical data and paraffin tissue blocks available for immunohistochemistry of TS, TP, and ERCC1 in this analysis. The median duration of follow-up was 41 months, with a range of 18–70 months. Our study protocol was approved by the Institutional Review Board of Samsung Medical Center.

### Treatment

Patients received cisplatin (60 mg m^–2^ intravenously on day 1) and capecitabine (1250 mg m^–2^ per dose orally twice a day on days 1–14). Treatment cycles were repeated every 3 weeks until documented disease progression, unacceptable toxicity, or patient refusal. The treatment response was evaluated every two cycles by RECIST, version 1.0.

### IHC staining and scoring

Tumour specimens from patients who were diagnosed on the basis of histological samples were evaluated for biomarker analyses. Full specimen sections (4 *μ*m thick) were cut from paraffin blocks and mounted on adhesive-coated or charged glass slides. An IHC method was used to determine the level of protein expression for TS, TP, and ERCC1.

Immunohistochemical staining was performed using the streptavidin–biotin complex method and the TechMate 1000 automated staining system (DakoChemmate, Glostrup, Denmark). The primary antibodies and working dilutions used were as follows: TS mouse mAb (clone TS106/4H4B1, 1 : 50 dilution in Tris/EDTA buffer (pH 9.0); Zymed, San Francisco, CA, USA) and TP mouse mAb (PGF.44C, 1 : 100 dilution; NeoMarkers, Fremont, CA, USA). Deparaffinised sections were treated with 3% hydrogen peroxide in methanol for 10 min to inhibit endogenous peroxidase. Sections were immersed in 0.01 M citrate buffer (pH 6.0) and heated in a pressure cooker for 30 min. Sections were then incubated with primary antibody for 50 min at room temperature. Each section was treated sequentially with a biotinylated secondary antibody (antimouse immunoglobulin) and a streptavidin–peroxidase complex (DakoChemmate). 3,3′-Diaminobenzidine tetrahydrochloride was used as a chromogen, and Mayer's haematoxylin counterstain was applied. Experiments on negative controls (isotype-matched irrelevant antibody) were run simultaneously.

For ERCC1 detection, mouse monoclonal antibody ERCC1 (8F1; NeoMarkers) was used at a dilution of 1 : 200 overnight in a humidified chamber. The primary antibody was visualised with an avidin–biotin complex (ABC) system (Dako, Carpinteria, CA, USA). Slides were washed in TBS, the relevant biotinylated goat antimouse IgG diluted at 1 : 100 was added, and the slides were incubated for 20 min at room temperature. The sections were washed again in TBS and incubated for 10 min in a solution of streptavidin–ABC–horseradish peroxidase diluted at 1 : 100. Colour was developed by adding 3,3′-diaminobenzidine tetrahydrochloride (Immunotech, Cedex, France). Finally, the sections were counterstained with Mayer's haematoxylin.

**Figure 1:**
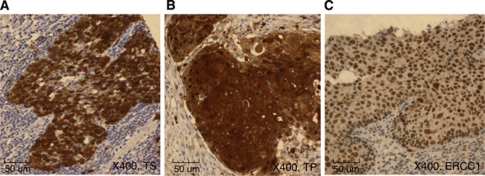
Immunohistochemical staining of TS, TP, and ERCC1 in oesophageal squamous cell cancer. (**A**) High staining intensity for TS. The TS staining was predominantly a cytoplasmic granular pattern ( × 400). (**B**) Positive sample with TP. The immunoreactivity was nuclear and cytoplasmic and it was highly expressed in mononuclear cells ( × 400). (**C**) High expression of ERCC1. The ERCC1 staining was strong and diffuse in the nucleus ( × 400).

For each patient, three full-specimen sections were analysed, one slide each for TS, TP, and ERCC1. The slides were assessed without the knowledge of clinical outcome by two experienced pathologists. Thymidine synthase and TP protein expression levels were detected in both the cytoplasm and nucleus, and ERCC1 showed nuclear staining (Figure 1).

The intensity and extent of the IHC staining was graded on a quantitative scale from 0 to 100% in proportion, except for ERCC1, and on a semiquantitative scale from 0 to 3, as follows: 0=no staining; 1=weak staining; 2=strong staining; and 3=very strong staining. The area of most intense staining was graded on a scale from 0 to 4, where 0=no staining, 1=0–10% staining of tumour cells, 2=10–25% staining of tumour cells, 3=25–50% staining of tumour cells, and 4=>50% staining of tumour cells. A TS score was calculated by multiplying the intensity and extent of grades. Thymidine synthase scores <6 were considered low, and a score >6 was defined as high in this study. The same criteria were applied for TP and ERCC1 staining results.

### Statistical analysis

The *χ*^2^ values of Fisher's exact tests, as indicated, were applied for the comparison of clinicopathological features of TS, TP, and ERCC1 tumours. Progression-free survival (PFS) was calculated from the first date of XP chemotherapy to the date of progression or death in the case of patients who died without progression. Overall survival (OS) was defined as the interval between the first date of chemotherapy and death or up to the date of the last follow-up evaluation. Both PFS and OS were calculated according to the Kaplan–Meier method. All reported *P-*values are two-sided. The contribution of prognostic variables to survival was analysed using the log-rank test for univariate analysis and the Cox proportional hazard model for multivariate analysis. The clinicopathological variables and expressions of TS, TP, and ERCC1 were examined for an association with the clinical outcome. The log-rank test was used to measure the association between TS, TP, and ERCC1, and survival. For quantitative analysis of TS and TP in terms of the extent of positive staining, the ANOVA test was used with response. We did not conduct survival analysis with quantitative results. Statistical significance was regarded as significant when *P*<0.05. All analyses were carried out using the Statistical Package for the Social Science (version17.0; SPSS Inc., Chicago, IL, USA) statistical software package.

## Results

### Patient characteristics and clinical outcome

Between July 2003 and December 2007, 113 patients with advanced oesophageal cancer received palliative chemotherapy at our institution, with XP combination chemotherapy as a first-line treatment. Among these 113 patients, tissue samples of 72 patients were available for analysis of IHC staining of TS, TP, and ERCC1. The patient baseline characteristics are listed in Table 1. All patients had histologically proven SCCA of the oesophagus, and no patient had adenocarcinomas of the gastro-oesophageal junction. The median age was 62 years, with a range of 31–72 years. In all, 25 (34.7%) patients received adjuvant 5-FU and cisplatin (FP) chemotherapy after surgical resection; 68 (94.4%) patients had an Eastern Cooperative Oncology Group performance status of 0–1. The sites of metastasis included the lymph nodes (76.4%), lung (26.4%), liver (18.1%), pleura (11.1%), skin (6.9%), and bone (5.6%).

Of the 72 subjects who were considered assessable for response to therapy, 69 had an overall RR of 51.4%, with 2 complete responses and 35 partial responses (51.4%). Ten (14.5%) patients had stable disease, but 22 (30.6%) patients experienced clinical or radiographic evidence of disease progression. Three (4.2%) patients were not assessable because of loss to follow-up. The patient population had a median of four cycles of the XP regimen. The median time to PFS was 4.2 months (95% confidence interval (CI) 3.0–5.4 months), and the median survival was 12.0 months (95% CI 9.2–14.8 months). The median duration of follow-up was 41 months.

### IHC staining results of TS, TP, and ERCC1 expression

Table 2 shows the IHC results of TS, TP, and ERCC1 expression according to intensity and proportion. The number and percentage of patients who had low scores are shown.

### Relationship between treatment response and biological marker expression

**Figure 2:**
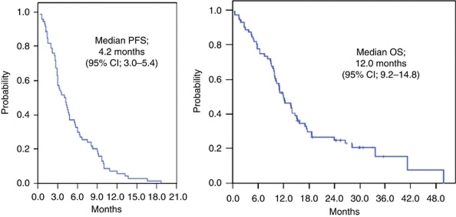
Kaplan–Meier curve for PFS and OS of XP in metastatic oesophageal SCCA.

**Figure 3:**
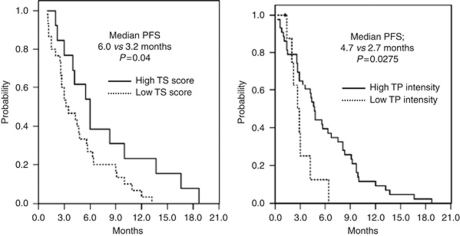
Thymidine synthase and TP expression and PFS.

**Figure 4:**
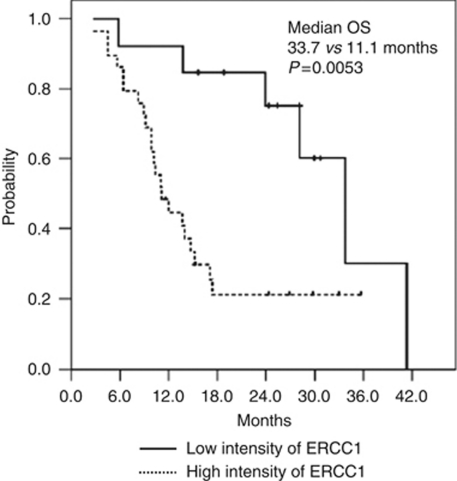
Excision repair cross-complementation group 1 expression and OS.

The association between TS, TP, and ERCC1, and response to XP, as measured by IHC, is listed in Table 3. A high TS proportion, high TS intensity, and high TS score predicted a response (TS<25% *vs* TS ⩾25%, RR 40.5 *vs* 59.5%, *P*=0.009; TS intensity 0–1 *vs* 2–3, RR 45.9 *vs* 54.1, *P*=0.030; TS score ⩽6 *vs* TS score>6, RR 40.5 *vs* 59.5%, *P*=0.005). High expression of TS and TP was also significantly correlated with treatment response to the XP regimen (high TS and TP *vs* low TS and TP, RR 54.1 *vs* 45.9%, *P*=0.022). High TS and TP expression with low ERCC1 expression (0–2 *vs* 3) showed a significantly better response compared with other levels of expression (high TS and TP and low ERCC1 *vs* other levels of expression, RR 63.2 *vs* 36.8%, *P*=0.016; Table 3, Figures 2, 3 and 4). In addition, quantitative analysis, as well as TS and TP extents, showed significant correlation with responses (RR 3.77, *P*<0.0001 for TS extent (%); RR 4.12, *P*=0.045 for TP extent by ANOVA test, Table 3).

### Univariate and multivariate analyses of factors associated with prognosis

We investigated the prognostic significance of various clinicopathological factors in patients with metastatic oesophageal SCCA. The univariate analysis showed that a high TS score, high TP score, and low ERCC1 expression were important factors affecting the prolongation of PFS and OS. Multivariate analysis was performed on capecitabine biological markers separately and with the platinum markers combined. Strong ERCC1 expression and a low TS score were identified as independent risk factors for PFS (hazard ratio (HR) 10.71, 95% CI 2.1–54.7, *P*=0.004 for strong ERCC1 expression; and HR 2.9; 95% CI 1.0–7.9, *P*=0.044 for a low TS score). Strong ERCC1 expression was the only unfavourable independent determinant for OS (HR 3.73; 95% CI 1.39–10.0; Table 4).

## Discussion

Thymidine synthase and TP have a key role in 5-FU resistance. Thymidine synthase, an essential enzyme needed for DNA synthesis, is the target enzyme of 5-FU. A number of studies have investigated TS expression and survival in colorectal cancer, but the data are conflicting (Popat *et al*, 2004). Some investigators have reported a survival benefit for patients with a high TS expression treated with 5-FU in adjuvant and metastatic colorectal cancer (Johnston *et al*, 1995; Edler *et al*, 2002; Soong and Diasio, 2005). In contrast to previous studies, different investigations have demonstrated a significant benefit in patients with low intratumoural TS levels (Allegra *et al*, 2002, 2003; Ciaparrone *et al*, 2006). Our results are consistent with previous reports and can be explained by the knowledge that high TS levels are most commonly associated with a responsiveness to 5-FU in patients with advanced disease. Although patients in this study were not randomly allocated to the study, exploratory analysis showed that the HR associated with capecitabine chemotherapy was 6.67 (95% CI 1.69–25.0) for advanced oesophageal SCCA with a high TS score (>6) compared with a low TS score.

Thymidine phosphorylase is the marker of a more aggressive and malignant tumour phenotype that has increased resistance to cytotoxic agents because of the loss of apoptotic potential. This study also examined the impact of TP expression and its correlation with prognosis. Our study demonstrated a significant association between TP expression and clinical outcome. Thymidine phosphorylase expression was shown to be a positive predictive factor for FU response. However, the prognostic role of TP is not clear. Conflicting results have been reported in different studies. Li *et al* (2004) evaluated the association between TP-messenger RNA expression in tumour tissue and some prognostic factors (grade, tumour size, lymph node status, phase S cellular fraction, ploidy, and clinical variables) with no statistically significant correlation. Yang *et al* (2002) analysed the expression of TP in 182 breast cancer patients with evidence of an association between TP expression, low grade, and low p53 expression. Thymidine phosphorylase-positive tumours seem to have a longer time to progression and OS, with a limited effect on angiogenesis. In contrast with these results, Tominaga *et al* (2002) published a retrospective study on TP expression in 650 samples of breast cancer. This study was not consistent with a previous report of an increase in tumour response and prolongation of OS in patients treated with FU and/or cisplatin-based chemotherapy for gastrointestinal cancer.

Excision repair cross-complementation group 1, the structure-specific DNA repair endonuclease responsible for 5′ incision, has a key role in the removal of adducts from genomic DNA through the NER pathway. We found that low expression of ERCC1 correlated significantly with a good response to systemic chemotherapy in patients with metastatic oesophageal SCCA. Kim *et al* (2008) reported that the low expression of ERCC1 correlated significantly with a good response to preoperative chemoradiotherapy in patients with oesophageal cancer, suggesting that ERCC1 expression may determine that the benefit of preoperative chemoradiotherapy may be more effective than immediate surgery in patients with a low ERCC1 expression. In contrast, immediate oesophagectomy may be as effective as preoperative chemoradiotherapy in patients with a high ERCC1 expression. These results were consistent with a prospective study in non-small-cell lung cancer, which showed that adjuvant platinum-based chemotherapy, compared with observation, significantly prolonged survival among patients with ERCC1-negative tumours (Olaussen *et al*, 2006). Jun *et al* (2008) compared their study of head and neck cancer treated with cisplatin-based concurrent chemoradiation with a study of non-small-cell lung cancer and showed that the proportion and pattern of ERCC1 expression varied according to tumour type. Multivariate analysis revealed that low expression of ERCC1 was an independent factor associated with a lower risk of cancer death. This result was also consistent with a previous report of an increase in tumour response and prolongation of OS in patients with oesophageal SCCA treated by cisplatin-based chemotherapy (Warnecke-Eberz *et al*, 2004; Joshi *et al*, 2005). Out data support the hypothesis that enhanced DNA repair decreases the benefit of platinum-based treatment.

If the combination of these three markers reflects the response and toxicity of the XP regimen in patients with metastatic oesophageal SCCA, we can predict who benefits from this regimen. As only a relatively small proportion of patients seem to benefit from systemic chemotherapy in metastatic oesophageal SCCA, considerable effort has been directed towards finding biomarkers that can accurately predict tumour response. Currently, however, there is insufficient evidence to justify the incorporation of these or any other candidate predictive markers into routine clinical practice for the selection of oesophageal cancer patients to receive XP. If one could identify the patients likely to respond to a particular regimen, therapy could subsequently be tailored accordingly and the survival rate could be improved.

The limitation of this study included the inherent weakness of IHC staining, such as its semiquantitative nature, different antibodies used in various studies, inter or intraobserver variation, the variable cutoffs for TS, TP, and ERCC1 positivity, and the effects of tissue ageing. In addition, the number of patients was relatively small, and the study was retrospective in design. These correlative studies must be viewed as hypothesis generating and interpreted with caution because of the small sample sizes and limitation of a retrospective study.

In conclusion, this study has demonstrated that TS, TP, and ERCC1 expressions show promising markers for response and survival prediction of XP chemotherapy in patients with recurrent or metastatic oesophageal SCCA. Further prospective randomised studies are needed to confirm our results.

## Figures and Tables

**Table 1:** Patients’ characteristics (*n*=72)

	**Number of patients (*n*=72)**	**%**
*Sex*		
Male	69	95.8
Female	3	4.2
Age, median (range)	62 (37–72)	
		
*Performance status*		
0–1	68	94.4
2	4	5.6
Previous adjuvant FP chemotherapy	25	34.7
XP cycle, median (range)	4 (1–12)	
		
*Metastatic sites*		
Lymph nodes	55	76.4
Lung	19	26.4
Liver	13	18.1
Pleura	8	11.1
Skin	5	6.9
Bone	4	5.6

Abbreviations: FP=fluouracil and cisplatin; XP=capecitabine and cisplatin.

**Table 2:** TS, TP, and ERCC1 by IHC staining

	**Number of patients with low scores**	**%**
*TS*		
Area (0–2 *vs* 3–4)	38/69	55.1
Intensity (0–1 *vs* 2–3)	40/69	58.0
		
*TP*		
Area (0–2 *vs* 3)	23/72	31.9
Intensity (0–2 *vs* 3)	3/72	4.2
		
*ERCC1*		
Area (0–2 *vs* 3)	22/41	53.7
Intensity (0–2 *vs* 3)	14/41	34.1

Abbreviations: ERCC1=excision repair cross-complementation group 1; IHC=immunohistochemical; TP=thymidine phosphorylase; TS=thymidine synthase. Score means multiplying the intensity and extent grades of the IHC.

**Table 3:** Association between TS, TP, and ERCC-1 and response (semiquantitative and quantitative analysis)

	**Response (CR+PR)**	
	***χ* ^2^**	**Relative risk, 95% CI**
TS extent (3–4 *vs* 0–2)	59.5 *vs* 40.5% (*P*=0.009)	3.70 (1.37–10.0)
TS intensity (2–3 *vs* 0–1)	54.1 *vs* 45.9% (*P*=0.030)	3.03 (1.10–8.33)
TS score (extent × intensity) (> 6 *vs* 6 ⩽)	59.5 *vs* 40.5% (*P*=0.005)	6.67 (1.69–25.0)
High TS, TP expression (both high score *vs* others)	54.1 *vs* 45.9% (*P*=0.022)	3.70 (1.23–11.11)
High TS, TP expression with low ERCC1 expression	63.2 *vs* 36.8% (*P*=0.016)	3.45 (1.33–11.10)
	**Response (CR+PR)**	
	**ANOVA**	**Relative risk**
TS extent (%)	*P*<0.0001	3.77
TP extent (%)	*P*=0.045	4.12

Abbreviations: ANOVA=analysis of variance; CI=confidence interval; CR=complete response; ERCC1=excision repair cross-complementation group 1; PR=partial response; TP=thymidine phosphorylase; TS=thymidine synthase.

Intensity was graded on a semiquantitative scale from 0 to 3, where 0=no staining, 1=weak staining, 2=strong staining, and 3=very strong staining. The area of the most intense staining was graded on a scale from 0 to 4, where 0=no staining, 1=staining of 0–10% of tumour cells, 2=10–25% of tumour cells, 3=staining of 25–50% of tumour cells, and 4=staining of >50% of tumour cells. A TS score was calculated by multiplying the intensity and extent grades. TS scores <6 were considered low, and score >6 were defined as high in this study. The same criteria were applied for TP and ERCC1 staining results.

**Table 4:** Multivariate Cox-regression hazard model for PFS and OS

	**PFS**				**OS**			
	***P-*value**	**HR**	**95% CI**		***P-*value**	**HR**	**95% CI**	
Low TS score	0.044	2.87	1.03	7.99	0.356	1.22	0.38	3.46
Strong ERCC1	0.004	10.71	2.10	54.71	0.009	3.73	1.39	10.04
Hepatic metastasis	0.118	2.57	0.79	8.41	0.235	1.11	0.03	2.13

Abbreviations: CI=confidence interval; ERCC1=excision repair cross-complementation group 1; HR=hazard ratio; OS=overall survival; PFS=progression-free survival; TS=thymidine synthase.
